# Status of Recombinant Factor VIII Concentrate Treatment for Hemophilia A in Italy: Characteristics and Clinical Benefits

**DOI:** 10.3389/fmed.2019.00261

**Published:** 2019-12-03

**Authors:** Mario Schiavoni, Mariasanta Napolitano, Gaetano Giuffrida, Antonella Coluccia, Sergio Siragusa, Valeria Calafiore, Giuseppe Lassandro, Paola Giordano

**Affiliations:** ^1^Associazione per la Lotta alle Malattie Emorragiche e Trombotiche, Maglie, Italy; ^2^Internal Medicine and Medical Specialities, Haematology Unit, Department of Health Promotion, Mother and Child Care, Reference Regional Center for Thrombosis and Haemostasis, University of Palermo, Palermo, Italy; ^3^U.O.C. di Ematologia, A.O.U. Policlinico “Vittorio Emanuele”, Catania, Italy; ^4^U.O.C di Medicina Interna, Centro Emofilia e Coagulopatie Rare-Ospedale “I.Veris delli Ponti”, Scorrano-ASL, Lecce, Italy; ^5^U.O.C. di Ematologia, Università degli Studi, Palermo, Italy; ^6^Dipartimento di Scienza Biomedica e Oncologia Umana, Università degli Studi di Bari “Aldo Moro”, Bari, Italy

**Keywords:** Hemophilia A, recombinant Factor VIII products, pharmacokinetics, inhibitors, EHL-rFVIII

## Abstract

The current interest in recombinant factor VIII (rFVIII) products stems from the fact that they offer a technological solution to prolonging the half-life of and reducing the risk of formation of alloantibodies (inhibitors) against FVIII in treated patients with hemophilia A (HA). The Italian health care system has authorized the use of a wide range of rFVIII concentrates of the first, second, and third generation, as well as new innovative rFVIII preparates with an extended half-life (EHL) (Kogenate FS®-Bayer, belonging to the second generation and replaced since 2017 by a product consisting of the same modified molecule; because it is only available until the end of the current year, it will not be considered in this review). Some of these products have unique pharmacodynamic and pharmacokinetic (PK) profiles, including an EHL. The first-generation full-length rFVIII (FL-rFVIII), octocog alfa (Recombinate® Baxter/BIOVIIIx), although the oldest rFVIII product, has several desirable features. Third-generation products include two modified octocog alfa molecules (Advate®, Shire; Kovaltry®, Bayer) as well as the B domain-deleted rFVIII (BDD-rFVIII) moroctocog alfa (ReFacto®-Pfizer). The B domain-truncated (BDT-rFVIII) turoctocog alfa (NovoEight®, Novo Nordisk), the BDD-rFVIII simoctocog alfa (Nuwiq®, Kedrion), the single-chain BDT-rVIII lonoctocog alfa (Afstyla®, CSL Behring), and the BDD-rFVIIIFc efmoroctocog alfa (Elocta®, Sobi-Biogen) are new, innovative products. Simoctocog alfa, because its peculiarities, is considered a fourth-generation rFVIII concentrate. Turoctocog alfa, simoctocog alfa, and lonoctocog alfa have a high affinity for von Willebrand factor (vWF) that reduces renal clearance and prolongs the half-life of rFVIII. Efmoroctocog alfa, a first-in-class rFVIII-Fc fusion protein (rFVIIIFc), has a half-life 1.5–1.8 times longer than that of conventional plasma-derived FVIII (pd-rFVIII) and other rFVIII products. Clinical studies have evaluated the efficacy, safety, and inhibitor development of all these innovative concentrates in both previously treated (PTPs) and untreated patients (PUPs). This review considers the rFVIII products that are indicated for the treatment of patients with severe HA, focusing on those that are commercially available in Italy. Their PK characteristics, immunogenicity, and clinical benefits are discussed and compared.

## Introduction

The treatment of HA has dramatically improved since the 1990s, when the outbreak of blood-borne infections, mainly due to the human immunodeficiency and hepatitis viruses, caused high rates of death and co-morbidities in many patients (1). This worldwide situation led to the implementation of virus inactivation in the manufacture of pd-FVIII and rFVIII products, improving replacement therapy.

In addition, many HA patients develop inhibitors in response to FVIII injections. Thus, since 2008, the European Haemophilia Safety Surveillance System (EUHASS; www.EUHASS.org) has carried out safety monitoring of substitution therapies for patients with hemophilia (2).

All rFVIII products have an improved safety profile, as their production includes virus inactivation procedures similar to those used in pd-FVIII concentrates (in first-generation products, human albumin underwent pasteurization at 60°C for 10 h; a solvent/detergent (S/D) step and nanofiltration were employed in the second and third generations, and chromatographic purification techniques were also performed) and furthermore the progressive removal of human and animal proteins from the final formulation. The different generations of rFVIII products reflect the evolution of the methods used in their manufacture. First-generation rFVIII production methods include the use of animal proteins in the cell culture medium and the addition of human serum albumin to stabilize the recombinant product; the culture medium of second-generation rFVIII contains human- or animal-derived proteins but not human albumin; finally, the most recent, third-generation rFVIII products have no added human- or animal-derived proteins at all.

For patients with severe HA, prophylaxis with FVIII concentrates is the main treatment regimen to reduce joint bleeding and other types of hemorrhage, thereby improving the health-related quality of life (HRQoL) of both patients and their caregivers (3, 4). Moreover, prophylaxis reduces inhibitor formation, which is the major adverse event of hemophilia treatment (5).

HA prophylaxis usually consists of intravenous FVIII injections every other day or three times per week due to the short half-life of FVIII of approximately 12 h (6). However, the need for frequent injections is a considerable burden for patients and their caregivers, particularly in children and in those patients with poor venous access. The development of EHL rFVIII products has slightly improved the management of these patients by allowing a reduction in the dose frequency, which in turn has increased adherence to treatment (7).

This review summarizes the characteristics of the rFVIII products currently available in Italy for the treatment of severe HA (Table 1). The potential clinical benefits of these concentrates, as well as safety concerns including high titer inhibitor formation, are discussed.

**Table 1:** Panel of recombinant factor VIII (rFVIII) products currently available in Italy.

**Molecule**	**Brand**	**Protein structure**	**Manufacturer/distributor**	**Cell line**
First-generation rFVIII				
Octocog Alfa	Recombinate^®^	Full-length rFVIII + albumin+pegylation	Baxter/Bioviiix	Chinese hamster ovary (CHO)
Third-generation rFVIII				
Octocog alfa	Advate^®^	Full-length rFVIII; albumin-free	Shire	CHO
Moroctocog Alfa	ReFacto AF^®^	BDD rFVIII; albumin-free	Pfizer	CHO
Innovative third-generation rFVIII				
Octocog alfa	Kovaltry^®^	Full-length rFVIII (glycosylation sites, sulfation of tyrosine sites)	Bayer	Baby hamster kidney
Turoctocog alfa	NovoEight^®^	BTD rFVIII (glycosylation sites, sulfation of tyrosine sites)	Novo Nordisk	CHO
Simoctocog Alfa	Nuwiq^®^	BDD rFVIII	Octapharma/Kedrion	Human embryonic kidney (HEK)
Lonoctocog alfa	Afstyla^®^	Single-chain rFVIII BDT-rFVIII	CSL-Bhering	CHO
Extended half-life rFVIII				
Efmoroctocog alfa	Elocta^®^	BDD rFVIII-Fc fusion	Biogen Inc./Sobi	HEK

### First-Generation Product

#### Recombinate®

Recombinate® (octocog alfa, Baxter Biotech; distributed in Italy by BIOVIIIx) is the only one of two first-generation rFVIII concentrates that is still commercially available in Italy. It is derived from a conditioned medium of chinese hamster ovary (CHO) cell cultures transfected with cDNAs for FVIII (8–10). Human albumin, polyethylene glycol (PEG) 3350 (3 mg/ml), sodium chloride, calcium chloride, and histidine are added as stabilizers. The product currently used in Italy is manufactured in Belgium and does not contain polysorbate 80. The co-expression of recombinant vWF with rFVIII contributes to the stabilization of the product.

Beginning in 1990, a multicenter, multinational, prospective clinical trial of Recombinate® was conducted on PUPs with severe/moderate HA (baseline FVIII ≤2%) in order to evaluate the safety and efficacy of the product, including the development of inhibitors (11). Of the 79 PUPs enrolled, 76 received at least one infusion of the concentrate, and the 72 patients (91%) who continued in the study were tested for rFVIII inhibitors. Adverse events were reported after nine of the 12,156 rFVIII infusions administered to the cohort (0.074%), but none were defined as serious.

Of the 72 patients, 22 (30.5%) developed rFVIII inhibitors: a low titer ( ≤5 BU) was measured in 13 (59%) patients and a high titer (>5 BU) in 9 (40.9%) patients. In 12 of the 22 patients (54.5%), the inhibitors became undetectable, including in 11 patients in the low-titer group. At the end of the study, nine of the 72 patients (12.5%) still had a high titer of inhibitors.

Ewenstein et al., in a prospective pharmacovigilance study that considered the incidence of inhibitor development worldwide in patients treated with Recombinate®, demonstrated that the overall percentage was 11.9% (95% confidence interval [CI 5.05–28.0%]) for PUPs and 0.123% (CI: 0.030–0.512%) for PTPs. The incidence of high-titer inhibitors (>5 BU) was 5.96% (CI: 3.00–11.8%) for PUPs and 0.0554% (CI: 0.0113–0.271%) for PTPs (12).

A trial comparing the PK of Recombinate® with that of pd-FVIII enrolled 69 PTPs with HA (67 with severe and two with moderate disease), who participated for a median of 3.7 (1.0–5.7) years with the aim of comparing the PK of rFVIII with that of pd-FVIII. The safety and long-term home-treatment efficacy of rFVIII was also assessed (13). At study entry, 44 patients were HIV seropositive and 25 were seronegative. Three patients had a history of inhibitors, but not at study entry. The results showed that the mean incremental recovery for rFVIII at baseline was 2.4%/IU/kg, essentially the same as that of pd-FVIII (2.5%/IU/kg). Furthermore, there was no significant change in recovery over a 30-month period. The mean *in vivo* half-life of rFVIII and pd-FVIII at baseline was the same: 14.7 h. However, over time, rFVIII demonstrated a statistically significant trend (*p* = 0.015) of a longer mean half-life, as determined at months 18 and 24 (Table 2).

**Table 2:** Time course of the half-life of rFVIII (Recombinate®) and a comparison with plasma-derived FVIII III (pdFVIII) [from (13)].

**Months**	***n***	**Half-life (h)** **(mean ± SD)**	**Range**	***P*-value** (vs. baseline)^*^
pd-FVIII	61	14.7 ± 5.1	5.8–40.8	1
rFVIII (Recombinate®) baseline	65	14.7 ± 4.9	6.8–34.7	–
3	58	15.3 ± 50	5.6–30.6	0.52
6	62	15.3 ± 5.0	6.4–27.5	0.47
9	12	16.7 ± 7.7	10.8–34.7	0.25
12	61	16.0 ± 8.7	8.1–60.2	0.32
15	11	16.9 ± 8.5	10.7–39.1	0.41
18	55	18.4 ± 7.1	9.0–41.9	0.002
21	4	14.5 ± 3.3	11.0–17.5	0.93
24	10	18.1 ± 4.2	10.9–25.8	0.04
27	3	15.7 ± 5.1	10.9–21.1	0.74
30	48	15.2 ± 4.9	7.5–28.7	0.59

* *p = 0.015 (Kruskal–Wallis test)*.

The Evaluation Study on Prophylaxis: a Randomized Italian Trial (ESPRIT) compared prophylaxis with on-demand treatment with the aim of preventing joint bleeding and joint damage in children with severe HA (14). Forty of seventy-two eligible patients ranging in age from 1 to 7 years, completed the study. Twenty were randomly assigned to be treated prophylactically with Recombinate® at a dose of 25/IU kg three times a week on nonconsecutive days and nineteen on-demand with the same product at a dose ≥25/IU kg. The results showed that children randomized to the prophylaxis regimen had significantly fewer of all hemorrhagic events and joint bleeding episodes than patients in the on-demand group (*P* < 0.05). Of the five patients who developed inhibitors (12.5%), three were in the prophylaxis group and developed inhibitors after 24–48 exposure days (EDs). The remaining two patients, who were in the on-demand group, developed inhibitors at 20 and 2 EDs. No patient suffered from life- or limb-threatening bleeding or from hemorrhage that required hospitalization.

### Third-Generation Products

#### Advate®

In 2003, a plasma/albumin-free formulation of octocog alfa containing sucrose was introduced as the first third-generation rFVIII originating from its precursor Recombinate®. The adapted recombinant system used in the production of the rFVIII molecule includes culture medium free of human- or animal-derived additives. The CHO cell line co-expressing FL-FVIII and vWF has not undergone any further genetic manipulations, and the cDNA sequences encoding the recombinant proteins are identical to those used to obtain Recombinate®. Processing includes an S/D step, a viral inactivation that preserves the structural and functional integrity of the rFVIII molecule but inactivates lipid-enveloped infectious agents (15). Following the S/D step, the eluate is further purified using anion exchange chromatography. The newly introduced rFVIII, given the name Advate®, contains the following stabilizers and excipients: mannitol, trehalose, sodium chloride, histidine, Tris, calcium chloride, polysorbate 80, and glutathione.

The PK of Advate® has been evaluated in adults and children based on FVIII activity measurements in blood samples obtained up to 48 h following each infusion. The mean half-life values in adults and children differ as a function of patient age (Tables 3, 4).

**Table 3:** Studies of Advate ® [from (16)].

**Study patients (*n*)**	**Endpoints**
Pivotal (108)	Pharmacokinetics, efficacy, safety, and immunogenicity in PTPs
Continuation^*^ (82)	Long-term pharmacokinetics, efficacy, safety, and immunogenicity in PTPs who completed the Pivotal study
Surgery^*^ (59)	Efficacy and safety in PTPs undergoing surgical/invasive procedures
Pediatric (53)	Pharmacokinetics, efficacy, safety, and immunogenicity in 6-year-old PTPs
Japanese Registry (15)	Pharmacokinetics, efficacy, safety, and immunogenicity in PTPs in Japan

*PTPs, previously treated patients*.

* *Patients were eligible to participate in more than one study*.

**Table 4:** Pharmacokinetic parameters (mean ± SD) of ADVATE® by age group (data from product characteristics documentation).

**Pharmacokinetic parameter**	**Mean ± SD (*n* = 7)** ** (1 to <24 months)**	**Mean ± SD (*n* = 32)** ** (2 to <5 years)**	**Mean ± SD (*n* = 24)** ** (5 to <12 years)**	**Mean ± SD (*n* = 35)** ** (12 to <16 years)**
AUC0-t (IU*h/ml)	1,240 ± 330	1,164 ± 424	1,396 ± 506	1,300 ± 469
Incremental recovery at Cmax^a^ (IU/dL per IU/kg)	2.1 ± 0.5	1.8 ± 0.4	2.1 ± 0.6	2.1 ± 0.5
*t* $\frac{1}{2}$ (h)	8.7 ± 1.4	9.5 ± 1.8	11.2 ± 3.5	12.0 ± 2.9
Maximum plasma concentration post-infusion (IU/dL)	104 ± 27	91 ± 19	105 ± 34	103 ± 25
Mean residence time (h)	10.4 ±2.5	11.8 ± 2.8	14.3 ± 4.3	14.9 ± 4.6
Volume of distribution at steady state (dL/kg)	0.4 ± 0.1	0.5 ± 0.1	0.6 ± 0.2	0.6 ± 0.1
Clearance (mL/kg*h)	4.3 ± 1.0	4.8 ± 1.5	4.1 ± 1.5	4.2 ± 1.2

Advate® has been evaluated in 11 clinical trials in PTPs and in one trial in PUPs with severe to moderate HA (FVIII ≤ 2% of normal). The five completed clinical trials have demonstrated the safety and efficacy of Advate® in adult and pediatric PTPs, for both prophylactic and on-demand treatment regimens and for perioperative hemostatic coverage (Table 5) (17).

**Table 5:** Pharmacokinetic study in PTPs treated with ReFacto AF® as determined in a chromogenic assay (data from product characteristics documentation).

**Pharmacokinetic parameters**	**Mean**	**SD**	**Median**
AUC0-t (IU*h/ml)	19.9	4.9	19.9
t1/2 (h)	14.8	5.6	12.7
Clearance (ml/h·kg)	2.4	0.75	2.3
Mean residence time (h)	20.2	7.4	18.0
*In vivo* recovery (IU/dL/IU/kg)	2.4	0.38	2.5

A three-part study investigated the use of Advate® in 10- to 65-year-old PTPs without previous or detectable inhibitors. Fifty-six patients were randomized to Part 1 of the study, comparing the PK of Advate® with that of Recombinate®. Patients with severe or moderate HA (baseline FVIII ≤2%) received an initial infusion (50 ± 5 IU/kg). Bioequivalence between the older and newer products in terms of PK was demonstrated. Part 2 of the study evaluated the efficacy, safety, and immunogenicity of Advate® in 108 patients (baseline FVIII ≤2%) who followed a standard fixed prophylactic regimen over a period of at least 75 EDs. Of the 510 bleeding episodes that occurred during the study, 93% were resolved with 1–2 infusions of Advate®, with an excellent/good hemostatic response rate of 86%. Part 3 was a double-blind, randomized, cross-over comparison of the PK of pilot-scale-prepared Advate® with commercial-scale-prepared Advate®. The bioequivalence of the products was demonstrated (18). The long-term efficacy and safety of Advate® in PTPs with severe or moderate HA (baseline FVIII ≤2%) and no previous or detectable FVIII inhibitors were shown in an open-label, two-part continuation trial of patients who completed the pivotal phase III Advate® study (19). An integrated analysis of the safety and efficacy of Advate® examined the results of six previously conducted clinical prospective studies in a total of 234 patients with HA (FVIII ≤2%) (median age 14.7 years; range: 0.02–72.7 years) and concluded that Advate® was safe and effective. One PTP developed an inhibitor, but there were no other safety concerns (20).

In a clinical trial that enrolled 55 PUPs (defined as having had up to three exposures to an FVIII product at the time of enrollment), 16 (29.1%) patients who received Advate® developed inhibitors: seven (12.7%) had high titers (>5 BU) and nine (16.3%) had low titers. Inhibitors were detected at a median of 13 EDs (min-max: 6–26 EDs) (16).

In all of the above-reported studies, no patient had to withdraw from a clinical trial due to an adverse reaction.

#### ReFacto AF®

ReFacto AF® (moroctocog alfa, Pfizer) is a bioequivalent albumin-free rFVIII produced from the second-generation product ReFacto®. It is manufactured using CHO cells grown in a chemically defined serum- and albumin-free cell culture medium that, per the definition of a third-generation product, contains no materials derived from human or animal sources. ReFacto AF differs from ReFacto® in that a synthetic peptide affinity ligand (TN8.2) replaces the murine monoclonal antibody used in affinity chromatography and a 35-nm viral filtration step is included in the purification process (21). Sucrose, calcium chloride dihydrate, L-histidine, sodium chloride, and polysorbate 80 are added as excipients.

Two studies evaluated the properties of Refacto AF® in 204 PTPs with severe/moderate HA (22). The objectives of the studies were similar: determinations of the PK equivalence of Refacto AF® vs FL-rFVIII (Advate®-Baxter) and the efficacy and safety of Refacto AF® during surgery. These studies used standard one-stage coagulation and chromogenic assays to measure FVIII levels. The results showed that BDD-rFVIII PK was equivalent to FL-rFVIII in 30 PTPs≥12 years of age (Table 5). In a cohort of PUPs, the PK of Refacto AF® was evaluated using a chromogenic assay. In both PTPs and PUPs, the PK of the product included its stability over long treatment times. Efficacy and safety were also demonstrated both in the prophylactic use of Refacto AF® and in on-demand treatment, including in patients with pre-existing target joints. Moreover, in all nine patients treated with Refacto AF® for surgical support, hemostasis was achieved with minimal blood loss ( ≤50 mL) and without the need for blood transfusions. Both studies confirmed the inhibitor safety of Refacto AF® and determined that the manufacturing modifications implemented in the new BDD-rFVIII albumin-free cell culture process were not associated with neo-antigenicity.

More recently, the United Kingdom Haemophilia Centre Doctors' Organization (UKHCDO) National Haemophilia Database identified a consecutive cohort of 103 PUPs with severe HA treated with ReFacto AF® (23). The study monitored time to inhibitor development and associated risk factors. Inhibitor development occurred in 35 patients (31.7%) prior to 50 EDs (*P*_(t≤50)_ = 0.33, [95% CI: 0.25–0.43]), including 15 (14.5%) with a high titer (≥5 BU; *P*_*e*(*t*≤50)_ = 0.16, [95% CI: 0.10–0.25]). The incidence of inhibitors was comparable to that in previously published studies of PUPs (24, 25). Anti-rFVIII antibodies were significantly associated with both high-risk mutations and a family history of inhibitors. Anti-CHO antibodies were detected in a low percentage of patients but were without a significant clinical effect. The authors concluded that, for the PUP cohort considered in the study, the proportion developing inhibitors was similar to in previous PUP studies of ReFacto AF® and comparable with PUP studies of other rFVIII concentrates; nevertheless, a formal comparison of the immunogenicity of factor VIII products would require a much larger sample.

#### Kovaltry®

Kovaltry® (octocog alfa, Bayer) is an unmodified Fl-rFVIII with an amino acid sequence identical to that of sucrose-formulated rFVIII (rFVIII-FS; Kogenate® FS, Bayer, Whippany, NJ, USA) but developed using innovative techniques (26). Kovaltry® and rFVIII-FS use the same cell expression system (baby hamster kidney [BHK] cells). The product includes the following excipients: 2.2% glycine, 1% sucrose, 30 mM sodium chloride, 2.5 mM calcium chloride, 20 mM histidine, and 80 ppm polysorbate 80. The production process incorporates two dedicated viral clearance steps: a detergent treatment step for inactivation and a 20-nm filtration step to remove viruses and potential protein aggregates. Compared with rFVIII-FS, Kovaltry® has glycans shifted to higher branched structures and a somewhat higher degree of sialylation. These modifications were added to improve the product's PK, specifically, giving it slower clearance, a higher area under the curve (AUC) value, and a longer half-life.

The PK of Kovaltry® was investigated in PTPs (0–61 years of age) with severe HA who were administered a dose of 50 IU/kg (Table 6). Kovaltry® was also extensively studied in the LEOPOLD clinical trials, comprising three open-label studies that enrolled patients of all age groups (children, adolescents, and adults) with severe HA. LEOPOLD I was a multi-center, open-label, cross-over, uncontrolled study conducted in adolescent and adult PTPs (age ≥12 years to <65 years) to evaluate the efficacy, safety, and PK of Kovaltry® in prophylaxis, and perioperative coverage. The prophylactic regimen (20–50 IU/kg two or three times per week) was assigned by the investigator based on the patient's individual requirements. The annualized bleeding rate (ABR) was the primary efficacy endpoint (27). LEOPOLD II was a multi-center, open-label, cross-over, uncontrolled, randomized study in adolescent and adult PTPs (age ≥12 years to <65 years) that evaluated the superiority of prophylaxis vs on-demand treatment with Kovaltry® over a 1-year treatment period. The prophylactic regimen was 20–30 IU/kg two times per week or 30–40 IU/kg three times per week. The treatment group was assigned by randomization. The primary efficacy endpoint was ABR (28).

**Table 6:** Pharmacokinetic parameters for Kovaltry® (50 IU/kg dose) as determined in a chromogenic assay (data from product characteristics documentation).

	**Patient age**			
**Pharmacokinetic parameter**	**0 to <6 years (*n* = 8)**	**6 to <12 years (*n* = 10)**	**12–17 years (*n* = 5)**	**≥18 years (*n* = 21)**
AUC [IU h/dL]	1544.7 ± 387.1	1214.5 ± 395.1	1572.0 ± 448.0	2103.4 ± 702.8
Cmax^a^ [IU/dL]	89.6 ± 27.4	81.6± 17.8	132.5 ± 46.3	133.1 ± 20.4
t$\frac{1}{2}$ [h]^b^	12.1 ± 2.7	12.0 ± 2.1	14.4 ± 5.5	14.2 ± 3.5
MRT IV^c^ [h]	17.7 ± 3.6	17.8 ± 2.9	19.8 ± 5.8	19.9 ± 4.9
Vss^d^ [dL/kg]	0.57 ± 0.13	0.79 ± 0.23	0.71 ± 0.39	0.50 ± 0.11
Clearance [dL/h/kg]	0.033 ± 0.009	0.045 ± 0.016	0.034 ± 0.010	0.027 ± 0.010

*AUC, area under the curve*.

a *Maximum drug concentration in plasma after a single dose*.

b *Terminal half-life*.

c *Mean residence time after i.v. administration*.

d *Apparent volume distribution at steady state*.

LEOPOLD KIDS (part A) was a multi-center, open-label, uncontrolled study of Kovaltry® in pediatric PTPs (age ≤12 years; ≥50 EDs). The PK, efficacy, and safety of routine prophylaxis and the perioperative management of bleeding were investigated. The primary efficacy variable was the ABR that occurred during routine prophylaxis within 48 h of the previous infusion. ABR during prophylaxis, independent of the time of infusion, was also analyzed. The prophylactic regimen was 25–50 IU/kg at frequencies of either two times per week, three times per week or every other day, adapted to the patient's need by the investigator. In LEOPOLD KIDS part A, Kovaltry® values for the maximum concentration and AUC tended to be lower in children than in adults. LEOPOLD-KIDS part B, a study in PUPs, is ongoing (29).

In all of the completed LEOPOLD studies, Kovaltry® was shown to be efficacious, whether for prophylaxis, on-demand treatment, or perioperative hemostasis. The effective prophylactic dose, defined as achieving a low ABR, was 20–40 IU/kg, given two or three times per week. The incidence of treatment-related adverse events was <7% across all LEOPOLD studies, and no PTPs developed inhibitors during the completed primary studies (30).

#### NovoEight®

NovoEight® (turoctocog alfa, Novo Nordisk) is produced from CHO cell lines without the use of human or animal plasma. Turoctocog alfa includes two amino acid chains, a heavy chain and a light chain, joined by non-covalent bonds. This activated glycoprotein has the same molecular structure as human FVIII and is subjected to post-translational changes that are similar to those of the plasma-derived molecule. The Tyr1680 (total native length) sulfation site, important for vWF binding, is fully sulfated in the turoctocog alfa molecule. The high affinity with vWF helps to reduce the renal clearance of the product and prolong its half-life. Sucrose, sodium chloride, L-histidine, polysorbate 80, L-methionine, calcium chloride dihydrate, sodium hydroxide, and hydrochloric acid are the excipients. Viral inactivation is ensured by detergent inactivation and a 20-nm nano-filtration step.

Three multicenter, open-label, uncontrolled studies were conducted to evaluate the safety and efficacy of turoctocog alfa in the prevention and treatment of bleeding in PTPs with severe HA (FVIII ≤1%). The studies included 213 patients: 150 adolescent and adult patients >12 years of age without inhibitors and with ≥150 EDs, and 63 pediatric patients <12 years of age without inhibitors and with ≥50 EDs. Of these 213 patients, 187 participated in the extension safety study. Treatment with turoctocog alfa was shown to be safe and effective for bleeding treatment and for prophylaxis in 14 patients undergoing a total of 14 surgical interventions, including 13 major and 1 minor operation; hemostasis was achieved in all surgical interventions, and no failure was reported.

The efficacy and safety of turoctocog alfa were evaluated in the guardian 1 trial (31). Its primary objective was to evaluate safety in adolescent and adult PTPs with severe HA, especially the incidence of FVIII inhibitor development. Other endpoints were efficacy during bleeding episode treatment, bleeding control, and ABR. The study concluded that turoctocog alfa was safe and well-tolerated in adolescent and adult patients with HA.

A multicenter, multinational, open-label, sequential dosing PK study comparing turoctocog alfa with Advate® was conducted in 23 patients with severe HA (32). The results of the primary PK were comparable between the two products. The 90% CI for the treatment ratio of Advate® to turoctocog alfa was within the acceptable bioequivalence interval of 0.8–1.25.

Guardian 3 evaluated the safety and efficacy of turoctocog alfa in pediatric patients 0–11 years of age with severe HA (FVIII <1%). Patients with >50 EDs to any FVIII product were included in the study (33). The primary objective and all of the efficacy endpoints were identical to those in guardian 1. No patients developed FVIII inhibitors during the study. Three serious adverse events occurred that were not related to the study medication: soft-tissue injury, viral gastroenteritis, and device-related infections. Overall, the authors concluded that turoctocog alfa is safe and well-tolerated in pediatric patients with HA.

A secondary endpoint in the guardian 1 and 3 studies was HRQoL related to the use of turoctocog alfa. Those results showed a fairly stable HRQoL for patients already treated with prophylaxis (34). The greatest improvement in HRQoL was seen in patients who switched from on-demand therapy to prophylaxis. Of the 13 surgeries (10 major, 3 minor) assessed throughout the guardian 1 and 3 studies, the hemostatic response achieved with turoctocog alfa was reported as excellent or good in all of them.

All patients in the guardian 1 and 3 studies or in phase 1 PK trials were eligible for participation in the extension study, guardian 2, which assessed the efficacy and safety of turoctocog alfa in prophylaxis or on-demand treatment in PTPs of all ages. The primary safety endpoint was the frequency of FVIII inhibitor development. Efficacy endpoints included ABR during prophylaxis, hemostatic response in the treatment of bleeds, and the number of injections required to treat bleeds. The results showed that the extended use of turoctocog alfa was safe and effective in the prevention and treatment of bleeding episodes in patients of all ages (35).

The clinical trial GUARDIAN^TM^ 4 will assess the efficacy and safety of turoctocog alfa in the prevention and treatment of bleeds in treatment-naïve HA patients (36). GUARDIAN™ 5 is assessing the safety and efficacy of turoctocog alfa in the long-term prevention and treatment of bleeding episodes. The PK parameters of turoctocog alfa in patients with severe HA are reported in Table 7.

**Table 7:** Pharmacokinetic profile of Turoctocog Alfa in PTPs with severe HA (coagulation assay) (data from product characteristics documentation).

	**Patient age**		
**PK parameters**	**0 to <6 years (*n* = 14)**	**6 to <12 years (*n* = 14)**	**≥12 years (*n* = 33)**
*In vivo* recovery (IU/ml)/(IU/kg)	0.018 (0.007)	0.020 (0.004)	0.022 (0.004)
AUC (UI*h)/ml)	9.92 (4.11)	11.09 (3.74)	15.26 (5.77)
Clearance (ml/h/kg)	6.21 (3.66)	5.02 (1.68)	3.63 (1.09)
t $\frac{1}{2}$ (h)	7.65 (1.84)	8.02 (1.89)	11.00 (4.65)
Vss (ml/kg)	56.68 (26.43)	46.82 (10.63)	47.40 (9.21)
Cmax (IU/ml)	1.00 (0.58)	1.07 (0.35)	1.226 (0.41)

*Mean values are reported*.

*Cmax, maximum drug concentration in plasma after a single dose; VSS, apparent volume distribution at steady state*.

#### Nuwiq®

Simoctocog alfa (Nuwiq®, Octapharma-Kedrion) is a BDD-rFVIII produced in genetically modified human embryonic kidney cells, rhFVIII, and so is defined as a fourth-generation rFVIII product (37, 38). It consists of a heavy chain followed by a 16-amino acid linker sequence and a light chain. The molecule is produced with human post-translational modifications but without human- or animal-derived materials being added to the serum medium. Because of its specific post-translational modifications, a decreased immunogenicity has been hypothesized. The excipients are sucrose, sodium chloride, calcium chloride bihydrate, arginine hydrochloride, sodium citrate bihydrate, and poloxamer 188. The purification process comprises five chromatographic steps, S/D treatment, and 20-nm nanofiltration in order to minimize the risk of pathogens in the final product.

Three studies (GENA 01, GENA 08, and GENA-03) were carried out in 135 PTPs ≥12 years (74 adult and 61 pediatric patients) with severe HA treated with simoctocog alfa for bleeding episodes or the prevention of bleeding during surgery (39, 40). In the 22 patients included in GENA-01, 91% of the 986 recorded bleeding episodes were resolved with one intravenous infusion of concentrate. Replacement therapy with simoctocog alfa was rated as excellent or good for 94% of the bleeding episodes. Hemostasis achieved with the preparate in the two surgeries that occurred during the study was also rated as excellent.

GENA 08 evaluated 32 patients age ≥18 years who were placed on standard prophylaxis with simoctocog alfa for the prevention or treatment of hemorrhagic events. Hemostatic prophylaxis during surgery was also determined (41). In patients receiving the product prophylactically, an average of 0.19 bleeds per month were recorded for each patient. Treatment with the concentrate was rated as excellent or good based on the control of the majority of episodes (81.5%). Almost all hemorrhages were resolved with a single injection. In the five surgeries carried out during the study, the drug was rated as excellent in preventing bleeding during four of the operations and moderately efficacious in one.

GENA-03 evaluated the efficacy of simoctocog alfa in the treatment of bleeding episodes in 59 children 2–12 years of age. Resolution in 81% of the bleeding episodes was achieved with one or two injections (42). The efficacy of prophylaxis vs on-demand treatment with simoctocog alfa in previously treated adults with severe HA was investigated in an indirect analysis comparing the results of the GENA-01 (on-demand arm) and GENA-08 (prophylaxis arm) trials. The superiority of prophylaxis (ABR 2.3; CI: 1.5–3.4) over on-demand treatment (ABR 57.7; CI: 43.3–76.9) was shown.

Prospective non-registrative trials, such as the extension studies GENA-11 (extension of GENA-01) and GENA-13 (extension of GENA-03) and the new study GENA-09 and its extension GENA-04, have also been carried out (43). The PK profiles of simoctocog alfa with respect to prophylaxis, breakthrough bleeding, and surgical prophylaxis were investigated in 22 PTPs in a single center in Russia.

GENA-21 (NuPreviq), a prospective, multicenter, open-label, phase III study evaluating PK-guided prophylaxis vs on-demand treatment with simoctocog alfa in 66 adult PTPs with severe HA, was recently completed. Personalized prophylaxis with simoctocog alfa was shown to be a more convenient treatment in the 58% of patients who were able to extend the injection intervals from three times weekly to twice weekly or less. In addition to less factor consumption, a lower ABR was recorded in this group (44).

To assess the efficacy of simoctocog alfa with respect to surgical prophylaxis, an integrated analysis of five studies (GENA-01, GENA-03, GENA-04, GENA-08, and GENA-09) was carried out due to the limited number of PTPs enrolled in each study. Overall, 19 patients underwent 34 surgical procedures (20 minor and 14 major). Drug efficacy was rated as excellent or good in 100% of the minor and 92% of the major operations. None of the patients developed inhibitors.

Finally, the GENA-05 (NuProtect) trial evaluated the immunogenicity, safety, and efficacy of simoctocog alfa in PUPs with severe HA (45). Data from a preplanned interim analysis were analyzed for 66 PUPs with ≥20 EDs. The authors reported that 8 of the patients developed inhibitors after a median of 11.5 EDs (range 6–24); low-titer inhibitors were observed in 5 patients (4 transient). The cumulative incidence (CI) was 12.8% (4.5–21.2%) for high-titer inhibitors and 20.8% (10.7–31.0%) for all inhibitors. The median value of ABR was 0 for spontaneous bleeds and 2.40 for all other bleeding in the inhibitor-free period. Simoctocog alfa turned out to be effective, being excellent or good in treating 91.8% of the hemorrhagic episodes, and was also effective in surgical prophylaxis, being excellent or good for eight (89%) interventions and moderate for one (11%). No side effects or adverse reactions were reported (46). However, the PK of simoctocog alfa, in particular its half-life, was generally not superior to those of third-generation rFVIII concentrates. The PK of simoctocog alfa is reported in Table 8.

**Table 8:** Pharmacokinetic profile of Simoctocog Alfa (dose: 50 IU/Kg) in pediatric, adolescent, and adult PTPs with severe HA (chromogenic substrate assay) (data from product characteristics documentation).

	**Patient age**		
**Pharmacokinetic parameter**	**2–5 years** ** (*n* = 13)**	**6–12 years** ** (*n* = 12)**	**18–65 years** ** (*n* = 20)**
AUC (h*UI/ml)	11.7 ± 5.3	13.2 ± 3.4	22.6 ± 8.0
t 1/2 (h)	9.5 ± 3.3	10.0 ± 1.9	14.7 ± 10.4
IVR (%/UI/kg)	1.9 ± 0.3	1.9 ± 0.4	2.5 ± 0.4
Clearance (ml/h/kg)	5.4 ± 2.4	4.3 ± 1,2	3.0 ± 1.2

*Values reported are mean ± SD*.

#### Afstyla®

Lonoctocog Alfa (Afstyla®, CSL Behring) is a unique, single-chain rVIII molecule. It has a truncated B-domain and heavy and light chains covalently linked to form a stable and homogeneous compound. The molecule binds with high affinity to vWF, which results in its increased stability and protection from degradation. Lonoctocog alfa is produced by recombinant DNA technology in CHO cells. L-histidine, calcium chloride, sodium chloride, sucrose, and polysorbate 80 are the excipients. Safety from infective agents is ensured by chromatography, S/D treatment, and serial nanofiltration.

Lonoctocog alfa was shown to be effective for prophylaxis, treatment, and the surgical management of bleeding episodes in PTPs with severe HA. Two prospective, non-comparative, multicenter trials (AFFINITY program) were carried out, one in children and the other in adolescents/adults. Eligible patients had been treated with an FVIII product for 50–150 EDs. The co-primary efficacy endpoints were the treatment success rate (excellent or good) and ABR in routine prophylaxis, which were compared with on-demand treatment (47). The three-part study included adolescent/adult patients ≥12–65 years of age. Part 1 evaluated the PK and short-term safety of a single dose of lonoctocog alfa. Patients who completed Part 1 participated in Part 2, which compared prophylaxis with on-demand treatment. Part 3 included 173 other PTPs, 146 of whom were treated on prophylaxis three times per week.

In the Part 1 study on 27 PTPs, the half-life of lonoctocog alfa was slightly longer (14.5 ± 3.8 vs. 13.3 ± 4.4 h) than that of Recombinate®, and clearance was 28–31% lower. In a *post-hoc* sub-analysis, the vWF antigen level for both products was shown to have a positive effect on half-life and a negative effect on clearance. This finding suggests that stronger binding to vWF improves the PK profile of FVIII concentrates. In pediatric PTPs, the half-life was shorter and the clearance longer than in adolescents and adults (48) (Table 9).

**Table 9:** Pharmacokinetic parameters in children, adolescents, and adults by age category following a single i.v. injection of 50 IU/kg of lonoctocog alfa (chromogenic assay) (data from product characteristics documentation).

	**Patient age**			
**Pharmacokinetic parameter**	**0 to <6 years** ** (*n* = 20)**	**≥6 to <12 years** ** (*n* = 19)**	**≥12 to <18 years** ** (*n* = 10)**	**≥18 years** ** (*n* = 81)**
IR (IU/dL)/(IU/kg)	1.60 (21.1)	1.66 (19.7)	2.00 (20.8)	1.69 (24.8)
Cmax (IU/dL)	80.2 (20.6)	83.5 (19.5)	106 (18.1)	89.7 (24.8)
AUC0-inf (IU*h/dL)	1,080 (31.0)	1,170 (26.3)	1,960 (33.1)	1,540 (36.5)
t1/2 (h)	10.4 (28.7)	10.2 (19.4)	14.2 (26.0)	14.3 (33.3)
Mean residence time (h)	12.4 (25.0)	12.3 (16.8)	20.4 (25.8)	20.0 (32.2)
Clearance (mL/h/kg)	5.07 (29.6)	4.63 (29.5)	2.90 (34.4)	3.80 (46.9)
Vss (mL/kg)	71.0 (11.8)	67.1 (22.3)	55.2 (20.8)	68.5 (29.9)

*Values are reported as the arithmetic mean, coefficient of variation [%]*.

*IR, incremental recovery; Cmax, maximum drug concentration in plasma after a single dose; VSS, apparent volume distribution at steady state*.

In Parts 2 and 3, the adult/adolescent patients were assigned to different prophylaxis regimens or on-demand therapy (20–40 IU/kg every second day or 20–50 IU/kg two to three times per week) (47, 49). In all prophylactic regimens, a low median ABR of 1.14 (Q1, Q3: 0.0, 4.2) and a median of spontaneous ABR of 0.00 (Q1, Q3: 0.0, 2.4) were recorded. The hemostatic efficacy was good or excellent based on a 93.8% control rate for bleeding episodes. The 13 patients who underwent 16 surgical procedures were given lonoctocog alfa for hemostatic prophylaxis. In the sub-analysis of perioperative management, the efficacy of lonoctocog alfa was rated as excellent or good in 100% of the procedures. Data on inhibitor incidence in PUPs participating in the ongoing CSL627 UNDERSCORE 3001 extension study are awaited.

### EHL rFVIII

#### Elocta®

Efmoroctocog alfa (Elocta®, Sobi-Biogen) is a first-in-class fusion protein consisting of a single molecule of human BDD-rFVIII covalently linked to the dimeric Fc domain of human immunoglobulin G1 (rFVIII-Fc). The excipients used are sucrose, sodium chloride, L-histidine, calcium chloride dihydrate, and polysorbate 20. The half-life of efmoroctocog alfa is about 1.5 times longer than that of conventional pd-FVIII and of rFVIII products.

The efficacy of efmoroctocog alfa in prophylaxis, treatment of acute hemorrhage, and perioperative management has been investigated in two open-label, non-comparative, multinational phase III trials enrolling PTPs ≥12 (A-LONG) or <12 (Kids A-LONG) years of age who had severe HA. Adults and adolescents were eligible for A-LONG if they had been treated with any FVIII product for ≥150 EDs and had a history of ≥12 bleeding events in the 12 months prior to the study. Children were eligible to enter Kids A-LONG if they had been treated with any FVIII product for ≥50 EDs (50).

The A-LONG and Kids A-LONG studies demonstrated the EHL of efmoroctocog alfa (1.4- to 1.8-fold longer than that of Recombinate®). These pivotal phase III trials also showed that the routine prophylactic administration of efmoroctocog alfa in PTPs (1–2 times per week in adults/adolescents ≥12 years of age; twice-weekly in children <12 years of age) was well tolerated, with no evidence of increased immunogenicity. In addition, efmoroctocog alfa was effective in preventing bleeding episodes, including in target joints. Prophylaxis with efmoroctocog alfa was also associated with maintained or increased physical activity in the majority of patients, including those with target joints at baseline. Recommended long-term prophylaxis consists of intravenous injections of efmoroctocog alfa every 3–5 days (51).

The ASPIRE extension study was completed in 2018. The final results demonstrated the safety and sustained efficacy of efmoroctocog alfa for up to 4 years in PTPs with severe HA. Data from the study also showed the safety and efficacy of efmoroctocog alfa in the parent trials with respect to the prevention and treatment of bleeding events, including in the surgical setting (52). No inhibitors, treatment-related serious adverse events or deaths were reported. The ABR remained low: with individualized rFVIII-Fc prophylaxis, the median spontaneous ABR across all of the studied age groups was <1 and the median spontaneous joint ABR was 0. Improved joint-health scores demonstrated a clinical benefit beyond the low bleeding rates. Both A-LONG and Kids A-LONG exclusively enrolled PTPs with severe HA. Since the cumulative risk of inhibitor development is higher in PUPs than in PTPs (~30 % vs. 2–3 per 1,000 patient-years), an open-label, multicenter, single-arm phase III study (PUPs A-LONG) is evaluating the safety and efficacy of efmoroctocog alfa in the prevention and treatment of bleeding episodes in PUPs <6 years of age who have severe HA. The primary endpoint is the development of inhibitors; secondary objectives are efficacy, factor consumption, and experience with immune tolerance induction in participants with inhibitors (53). Although susceptible to over- or under-estimation, an interim analysis of A-LONG PUPs is currently available in anticipation of the final data. Among the 95 enrolled PUPs, 89 received the studied treatment, of whom 20 were started on a prophylactic regimen and 69 on an episodic regimen. Among the latter, 50 patients were switched to prophylaxis, such that the prophylactic arm of the study consisted of 72 pediatric patients. The pre-planned PUP-A interim analysis, based on a data cut-off of 12 March 2018, showed that the study enrolled a high proportion of patients with a family history of inhibitors (20%), a known risk factor for inhibitor development. According to the primary analysis (68 patients with ≥10 EDs), inhibitors occurred in 30.9% of patients (*n* = 21); the rate of high-titer inhibitors was 14.7% (*n* = 10). High-titer inhibitors developed after up to 11 EDs (median 8; range 4–11) (54, 55). Efmoroctocog alfa prophylaxis was well-tolerated and efficacious, with an overall median ABR of 1.16 (0, 4.14) and no spontaneous joint bleeding. The PK profile of the product is reported in Table 10.

**Table 10:** Pharmacokinetic profile of efmoroctocog alfa (coagulation and chromogenic assays) (data from product characteristics documentation).

**Pharmacokinetic** ** parameters**	**ELOCTA^a^**	**ELOCTA^b^**
	**(*n* = 28)**	**(*n* = 27)**
AUC/dose (UI*h/dL per UI/kg)	51.2 (45.0–58.4)	47.5 (41.6–54.2)
Cmax (UI/dL)	108 (101–115)	131 (104–165)
Clearance (mL/h/kg)	1.95 (1.71–2.22)	2.11 (1.85–2.41)
t$\frac{1}{2}$ (h)	19.0 (17.0–21.1)	20.9 (18.2–23.9)
Mean residence time (h)	25.2 (22.7–27.9)	25.0 (22.4–27.8)
Vss (mL/kg)	49.1 (46.6–51.7)	52.6 (47.4–58.3)

*Values are reported together with their 95% confidence intervals*.

*VSS, apparent volume distribution at steady state*.

a *Coagulation assay*.

b *Chromogenic assay*.

Data on inhibitor frequency in PTPs and PUPs using all of the rFVIII products are reported in Table 11.

**Table 11:** Rate of total and high-titer inhibitors in PTPs and PUPs using rFVIII products in Italy.

**Recombinant product**	**Total incidence of inhibitors in PTPs (%)**	**Incidence of high-titer inhibitors in PTPs (%)**	**Total incidence of inhibitors in PUPs (%)**	**Incidence of high-titer inhibitors in PUPs (%)**
Recombinate® (Baxter-Bioviiix): first-generation full-length rFVIII	0.123^a^	0.55	30.5^a^	12.9
ReFacto AF® (Pfizer): third-generation BDD-rFVIII	**–**	**–**	33	14.5
Advate AF® (Bayer): third generation full-length rFVIII	**–**	**–**	29.1	12.7
Kovaltry® (Bayer): third-generation full-length rFVIII	0	0	Ongoing LEOPOLD KIDs (Part B) study	Ongoing LEOPOLD KIDs (Part B) study
Novoeight® (Novo Nordisk): third-generation BDT rFVIII	0	0	Ongoing guardian 4 study	Ongoing guardian 4 study
Nuwiq® (Octapharma-Kedrion): third-generation BDD-rFVIII	0	0	20.8	12.8
Afstyla® (CSL Behring): third-generation single-chain rFVIII	0	0	Ongoing CSL627 UNDERSCORE 3001 extension study	Ongoing CSL627 UNDERSCORE 3001 extension study
Elocta® (Biogen-Sobi): extended half-life BDD- rFVIII^b^	0	0	30.9	14.7^b^

*rFVIII, recombinant factor VIII; BDD, B-domain-deleted; PUPs, previously untreated patients; PTPs, previously treated patients*.

a *Including high titer, low titer, and transient inhibitors*.

b *Interim analysis of A LONG study PUPs*.

## Discussion

Following the outbreak in the 1980s of blood-borne infectious diseases (particularly HIV and HBV/HCV infections) in hemophilia patients who received pd-FVIII products that had not been subjected to virus inactivation or removal treatments, the manufacturers of those products introduced virus sterilization steps into their production methods and increased the purity of the preparations via DNA genetic engineering, thus greatly improving safety. All of the rFVIII products currently available in Italy have been tested in well-conducted clinical trials in which they were shown to be effective and safe. However, the ever-increasing purity of the molecule has made these preparations more susceptible to inhibitor development than was the case with pd-FVIII concentrates. Consequently, there is currently an urgent need to minimize the risk of inhibitor development linked to replacement treatment/prophylaxis with rFVIII products. This is especially the case for high-titer inhibitors, which, according to clinical studies carried out worldwide, mainly develop in PUPs after ≥ 25/50 EDs.

The first-generation rFVIII molecules Recombinate® and Kogenate® were introduced in Italy in the 1990s. Recombinate® is currently distributed in Italy by BIOVIIIx. The characteristic of the product that accounts for its continued use is its stabilization by human serum albumin and polyethylene glycol (PEG 3350). The covalent linkage of polyethylene glycol (PEGylation) to FVIII increases the molecular weight and size of the protein by surrounding it with a hydrophilic cloud. This molecular change is thought to have a protective effect, reducing the product's susceptibility to proteolytic activity and degradation. PEGylation also changes the surface charge of the protein, which may interfere with its clearance, as the half-life of PEGylated rFVIII is longer than that of non-PEGylated FL-rFVIII (56). In addition, co-transfection of rvWF with the rFVIII-encoding plasmid during the initial production process not only helps to stabilize Recombinate® but may also protect it from inhibitor development. The putative modulatory role of vWF in the development of inhibitors in patients with HA is being intensely investigated, and differences in immunogenicity between pd-FVIII and rFVIII concentrates have been attributed to vWF (57–61). These considerations may explain the trend of Recombinate® to show a longer half-life over time (Table 2) and a similar or lower rate of high-titer inhibitors in PUPs (11, 62) (Table 11). Moreover, the absence of polysorbate 80 in the concentrate improves the safety of the product (63). In PUPs and PTPs treated with Recombinate® over many decades, there have been no adverse reactions or side effects attributable to continuous exposure to the excipient PEG, despite a study showing its potential accumulation or damage (e.g., vacuolation in epithelial cells of the choroid plexus) in tissues (64).

Advate® (octocog alfa), a third-generation, human-protein-free FL-rFVIII, is derived from Recombinate® and has similar efficacy, safety, and immunogenicity in PTPs and PUPs with severe HA. However, Advate® and other rFVIII products differ from Recombinate® in that their excipients include polysorbate 80.

The BDD-rFVIII ReFacto AF® is an innovative structural modification of FVIII derived from ReFacto®. Its efficacy is equivalent to that of FL-rFVIII products. The immunogenicity of ReFacto AF® in PUPs was evaluated in a study conducted by UKHCDO. The results showed a slightly high incidence of high-titer inhibitors (14.5%) in PUPs.

Kovaltry®, a derivative of Kogenate FS®, is an unmodified FL-rFVIII produced using a genetically engineered BHK cell line. Its post-translational modifications are similar to those of endogenous FVIII, including glycosylation and tyrosine sulfation, which account for the high affinity of Kogenate FS® for vWF, in turn slightly prolonging its half-life. The results from the Leopold study confirmed the efficacy and safety of Kovaltry® for twice/thrice weekly prophylaxis, on-demand treatment, and the control of perioperative bleeding in patients of all ages. Moreover, at least within the completed primary studies, no FVIII inhibitors developed in PTPs. Anti-HSP70 antibodies were detected in some patients but were without any clinical effect.

Turoctocog alfa-rFVIII (Novoeight®) was the first BDT-rFVIII. Its high affinity for vWF accounts for its slightly longer half-life. The guardian study of a large population of PTPs with severe HA demonstrated that turoctocog alfa is effective and safe in preventing and treating bleeding, is well tolerated, and is not associated with unexpected safety issues or FVIII inhibitor development. A PK study comparing turoctocog alfa with Advate® showed that the primary PK parameters of the two products were comparable.

Of particular interest is the BDD-rFVIII simoctocog alfa (Nuwiq®), the first product obtained from a human embryonic kidney cell line. The GENA study and its extensions demonstrated that simoctocog alfa was a good/excellent rFVIII in adult and pediatric PTPs, whether for prophylaxis, on-demand treatment, or intraoperative bleeding prevention. Definitive data on inhibitor development in a large population of PTPs enrolled in various prospective trials, as well as preliminary data from the NuProtect study in PUPs, suggest a low inhibitor rate in patients treated with simoctocog alfa.

Lonoctocog alfa (Afstyla®) is a unique single-chain rFVIII. Its safety, efficacy, and PK profile were evaluated in two open-label studies, one in children and the other in adolescents/adult populations. The results of those studies showed good/excellent hemostatic efficacy both in the control of episodic hemorrhagic events and in bleeding prophylaxis. In addition, the adolescent/adults study demonstrated hemostatic efficacy during the perioperative management of bleeding in patients undergoing surgical procedures. The high affinity of lonoctocog alfa for vWF results in its stabilization and reduced clearance of rFVIII molecules. Data on inhibitor incidence in PUPs from the ongoing CSL627 UNDERSCORE 3001 extension study are not yet available.

Efmoroctocog alfa (Elocta®), a BDD-human r-FVIII covalently linked to the Fc domain of human IgG1, is the only EHL rFVIII commercially available in Italy. The half-life of efmoroctocog alfa is 1.5–1.8 times longer than that of other, conventional rFVIII products (19 h and 12–14 h in patients ages ≥12 and <12 years, respectively). Data from pivotal phase III studies (A-LONG in adults and adolescents ≥12 years of age; Kids A-LONG in children <12 years of age) and an extension study (ASPIRE) demonstrated the long-term effectiveness of efmoroctocog alfa for the treatment of acute bleeding episodes, perioperative management and routine prophylaxis in PTPs with severe HA.

Routine prophylaxis consisting of intravenous injections every 3–5 days is recommended, although once weekly may be suitable for some patients. Neither inhibitor development nor severe adverse events have been reported. Preliminary data from an interim analysis of the PUPs A-LONG ongoing study now available would confirm the hemostatic efficacy of efmoroctocog alfa. The cumulative incidence of inhibitor development is 30.9% while the rate of high-titer inhibitor development is 14.7%. Efmoroctocog alfa used prophylactically is well-tolerated and efficacious, with a very low median ABR overall and no spontaneous joint bleeding.

## Conclusion

Currently, the National Health Care System in Italy guarantees the availability of rFVIII products for the prophylaxis and episodic treatment of patients with severe HA. Third-generation rFVIII concentrates represent a wide range of high-quality products that offer efficacy and safety in all therapeutic regimens. Today, with the availability of EHL-rFVIII, the quality of life of both patients and caregivers is improving. Nonetheless, the first-generation rFVIII Recombinate®, far from being obsolete, continues to be effective and safe. The development of inhibitors, the most serious complication of replacement therapy, seems to be of very limited importance in PTPs who have switched to third-generation rFVIII products. In clinical studies, all of these newly developed concentrates have demonstrated efficacy and safety; nevertheless, they do not seem to be less immunogenic in PUPs than other rFVIII products. Despite the structural and functional improvements of new rFVIII concentrates, the incidence of high-titer inhibitors in PUPs remains a very significant and still unsolved problem.

## Author Contributions

All authors wrote and revised the final version of the manuscript.

### Conflict of Interest

The authors declare that the research was conducted in the absence of any commercial or financial relationships that could be construed as a potential conflict of interest.
